# Effects of Gas and Steam Humidity on Particulate Matter Measurements Obtained Using Light-Scattering Sensors

**DOI:** 10.3390/s23136199

**Published:** 2023-07-06

**Authors:** Hyunsik Kim, Jeonghwan Kim, Seungjun Roh

**Affiliations:** 1 Department of Civil Engineering, Korea National University of Transportation, Chungju 27469, Republic of Korea; jeonghwan.kim@ut.ac.kr; 2 School of Architecture, Kumoh National Institute of Technology, Gumi 39177, Republic of Korea; roh@kumoh.ac.kr

**Keywords:** particulate matter, humidity, light-scattering, sensor

## Abstract

With the increasing need for particulate matter (PM) monitoring, the demand for light-scattering sensors that allow for real-time measurements of PM is increasing. This light-scattering method involves irradiating light to the aerosols in the atmosphere to analyze the scattered light and measure mass concentrations. Humidity affects the measurement results. The humidity in an outdoor environment may exist as gas or steam, such as fog. While the impact of humidity on the light-scattering measurement remains unclear, an accurate estimation of ambient PM concentration is a practical challenge. Therefore, this study investigated the effects of humidity on light-scattering measurements by analyzing the variation in the PM concentration measured by the sensor when relative humidity was due to gaseous and steam vapor. The gaseous humidity did not cause errors in the PM measurements via the light-scattering method. In contrast, steam humidity, such as that caused by fog, resulted in errors in the PM measurement. The results help determine the factors to be considered before applying a light-scattering sensor in an outdoor environment. Based on these factors, directions for technological development can be presented regarding the correction of measurement errors induced by vapor in outdoor environments.

## 1. Introduction

Particulate matter (PM) is an aerosol in a solid or liquid form that is known to have various harmful effects on the human body. Therefore, the importance of measuring PM concentrations has been increasingly emphasized [1,2,3,4]. The well-known methods used to measure PM concentrations are the gravimetric, beta-ray, and light-scattering methods. The gravimetric method is mostly used as a reference method for PM measurement because of its high accuracy [5]. However, it refers to offline manual filter sampling conducted for a long term (typically 24 h), and the measured data are usually not available for a while because of the weighing process [6,7]. The beta-ray method is also known as one of the highly accurate methods for PM measurement. It estimates PM mass using beta-ray energy attenuation across a PM filter, and this process takes a much shorter time compared to the gravimetric method. However, the time resolution still takes a few minutes, and the large size and high cost of the instrument are the weaknesses for wide application [7]. Each method has its own merits and demerits, but the most suitable method for real-time concentration monitoring is the light-scattering method [8]. In this method, light is irradiated to the airborne PM, such that the light is scattered by the PM. If the irradiation of light targets the PMs with identical physical properties, the amount of scattered light will be proportional to the mass concentration of the PMs; thus, the amount of scattered light can be measured and used to estimate the PM concentration [9,10,11]. This method allows for real-time monitoring, easy portability, and simultaneous measurements of particles such as PM_2.5_, PM_10_, and total suspended particles according to particle size using a single device. However, errors may occur when converting the particle number concentration into the mass concentration. Despite the risk of potential errors, a light-scattering PM sensor serves well as a simple and portable PM detector based on its ready portability. Notably, the demand for air quality monitoring techniques corresponding to the recent advancement of smart technology that can be applied in smart cities has been increasing. Consequently, PM sensors that can be employed in the light-scattering method for outdoor monitoring should be developed [12].

A greater number of factors should be considered for outdoor measurements of PM concentration than for indoor measurements. In particular, the characteristics of the light-scattering method help measure not only the airborne dust particles but also the vapor as an aerosol, thus posing a challenge to the accurate measurement of dust concentration outdoors, where the amount of vapor in the atmosphere fluctuates more frequently than that indoors. Hence, several previous studies have identified variations in relative humidity (RH) as a factor influencing the PM measurement obtained using light-scattering sensors; RH has been the main factor to consider when monitoring the PM under outdoor conditions [13,14,15,16,17,18,19,20,21,22]. However, RH is a concept indicating the concentration of vapor in a gaseous state in the atmosphere [23,24], and therefore, it theoretically cannot be the primary factor affecting the PM concentration in the light-scattering method, as the method counts the aerosols in solid and liquid states [25,26]. For an extremely high RH, the condensation of vapor on the airborne PM could affect the changes in PM concentration in physical terms, and if the vapor is not in a gaseous state but a state of liquid aerosol (or steam), such as fog, it could be the target of light-scattering sensors [27]. However, most previous studies have not been able to clearly distinguish the light-scattering effect according to the variation in the state of the ambient humidity and report the findings that solely reflect the changes in RH.

Thus, to determine the effects of humidity on the PM measurements obtained using light-scattering sensors, an empirical study was conducted to analyze the variations in the PM concentration measured after supplying gaseous and steam vapor. Consequently, the trend of the variations in the PM concentration measured by the sensor was examined for an increase in RH caused by gaseous vapor and steam vapor. Ultimately, based on the results, the factors to be considered for applying light-scattering sensors to measure PM concentration under outdoor conditions were discussed.

## 2. Materials and Methods

**Figure 1:**
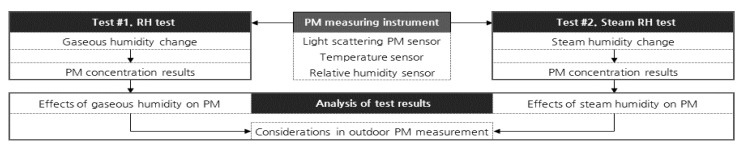
Research framework.

In this study, two independent tests were conducted to identify the effects of gaseous and steam humidity on the measurements of PM using light-scattering sensors. First, an RH test was conducted to analyze the variations in the PM measurements owing to gaseous vapor with a direct impact on RH values. Second, a steam RH test was conducted to analyze the variations in the PM measurements owing to steam vapor with liquid-state particles. The PM sensor used in the tests was Cubic PM2009 (Cubic Sensor and Instrument Co. Ltd., Wuhan, China) with an IP65 level of dustproof and waterproof performance, which can be applied under outdoor conditions [28]. To apply the PM sensor, a measuring instrument was developed to measure the humidity and temperature with the PM concentration simultaneously. This instrument was a prototype, and its performance was experimentally validated in a prior test, i.e., “Certification of Simple Fine Dust Meter” based on the “Special Act on PM Reduction and Management” in South Korea [29,30]. The reliability of the instrument was thus verified via a certification test under low-RH conditions. The results of the two tests were analyzed to identify the effects of the state of ambient humidity on the PM measurements obtained using light-scattering sensors, and the considerations when applying the sensors in an outdoor environment were discussed. The relevant framework of this study is presented in Figure 1.

### 2.1. RH Test

**Figure 2:**
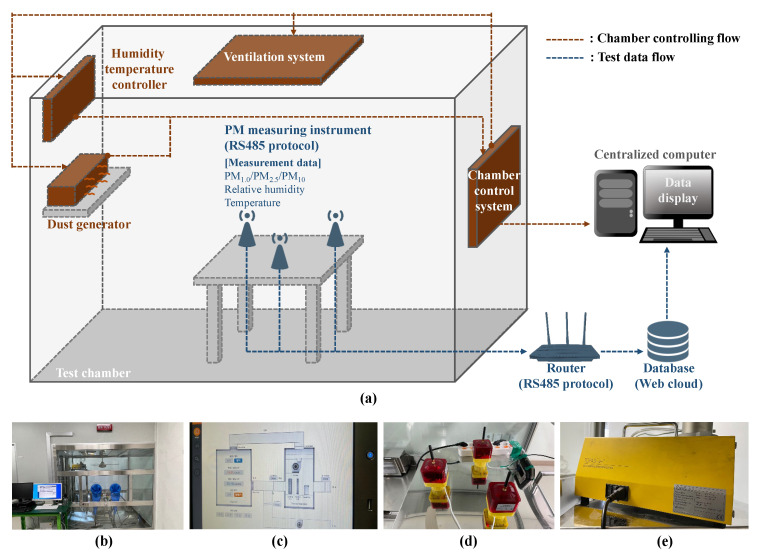
Details of (**a**) RH test procedure, (**b**) chamber, (**c**) chamber control system, (**d**) PM measurement instrument, and (**e**) dust generator.

The RH test was conducted under the conditions described in Figure 2 to measure the PM concentrations corresponding to a constant change in RH induced by the supply of gaseous vapor in a sealed chamber. In this manner, the effects on PM measurements related to RH were analyzed. The test was performed in a 30 m^3^ test chamber containing an electric heating and humidifying system that supported an all-in-one dust monitoring test. The test was conducted according to the “Test Method for Optically Equivalent PM Mass or Number Concentration Measurement using Low-Cost Dust Sensor” as a certified method for air purifiers in South Korea. For the generated particles, the A1 ULTRAFINE TEST DUST of ISO 12103-1 was used, and for the dust generator, SAG 410 of TOPAS was used [31,32]. In addition, the conditions inside the test chamber were such that the RH was altered by 10% each time under a constant temperature (24 ± 0.5 °C), while the concentrations of PM_1.0_, PM_2.5_, and PM_10_ were measured using the three pilot devices developed in this study. The sections below RH 30% were extremely dry regions with a negligible level of impact of RH and were thus removed from the scope of the test. In addition, the sections beyond RH 70% posed a difficulty in maintaining the inner chamber RH at a constant level; hence, the data collected from RH 30% to 70% were used, and the results from over RH 70% were excluded from the analyses.

The test procedure is summarized as follows: three measuring instruments for the test were selected at random and positioned at the center of the chamber. The chamber interior was thoroughly ventilated to ensure a state without PM, and the reference RH condition for the test to be performed in such a clean-state chamber was provided, while the generation of particles was controlled to reach the reference level of 50 μg/m^3^ which is the standard level of high PM concentration in Korea [33]. As PM particles sink in time to decrease the PM concentration inside the chamber, the PM concentration measurements for the first five minutes, which were obtained immediately after checking a stabilized level of 50 μg/m^3^ concentration of PM_2.5_ for each RH condition (i.e., five PM values per minute), were used as the data to be analyzed.

### 2.2. Steam RH Test

**Figure 3:**
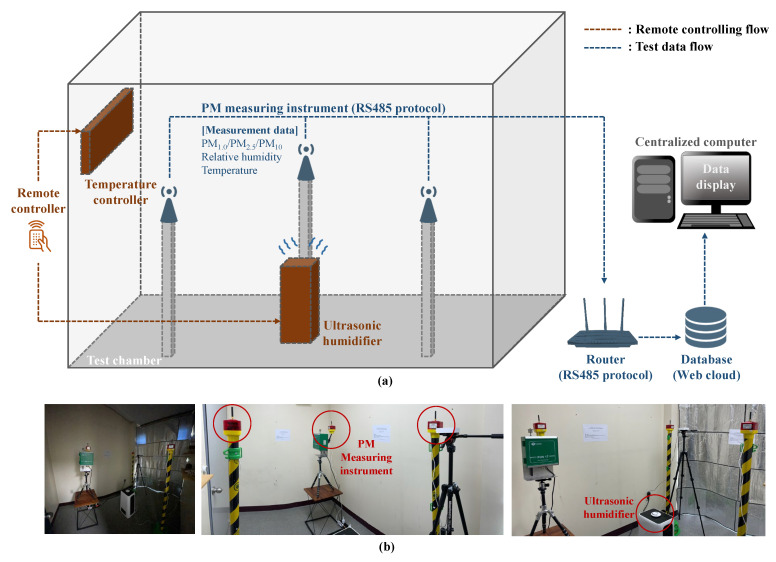
Details of (**a**) steam RH test procedure, and (**b**) instrument installation for steam RH test.

The steam RH test was conducted to analyze the effects of steam vapor, i.e., a liquid-state aerosol such as fog, on the measured PM concentrations in an outdoor environment. For this, three measuring instruments were installed inside a sealed lab at identical heights of 1.5 m, and an ultrasonic humidifier was installed at the center of the lab to supply steam. There are three broad types of humidifiers: vaporizing, heating, and ultrasonic. The vaporizing humidifier supplies a gaseous vapor, and the heating humidifier may affect the temperature change inside the lab. Therefore, the device selected for supplying steam was the ultrasonic humidifier [34,35,36]. The test was performed five times (RH 40–80%), as the reference RH with the operation of the humidifier was increased by approximately 10% each week. The lab conditions before the test were RH ≤ 40% with all three PM types, PM_1.0_, PM_2.5_, and PM_10_, below 10 μg/m^3^. A certain duration is required for the lab conditions to settle to a constant level immediately after the RH has been increased; therefore, the data for the first day of each week were excluded from the analyses. The sealed lab with the installed devices for the steam RH test is shown in Figure 3.

### 2.3. Analysis of Test Results

Based on the results of the RH test and steam RH test, the correlation between RH and PM concentration was analyzed to identify the effects of gaseous and steam humidity on PM measurements obtained using light-scattering sensors. For the correlation analysis, the Statistical Package for the Social Sciences (SPSS) software was used, and the trend of the optimal variations in PM concentrations for different RH sections in each test was analyzed. The results of the analyses were ultimately used for discussing the factors to be considered to monitor the PM concentrations under outdoor conditions.

## 3. Results and Discussion

### 3.1. RH Test Data

In the RH test, the three measuring instruments inside the test chamber measured the mean concentrations of PM_1.0_, PM_2.5_, and PM_10_ per minute for five minutes in the RH 30–70% range. The results are presented in Table 1. The actual data analysis generated decimal values, which are rounded off to the nearest integer in Table 1 for simplicity.

### 3.2. Steam RH Test Data

**Figure 4:**
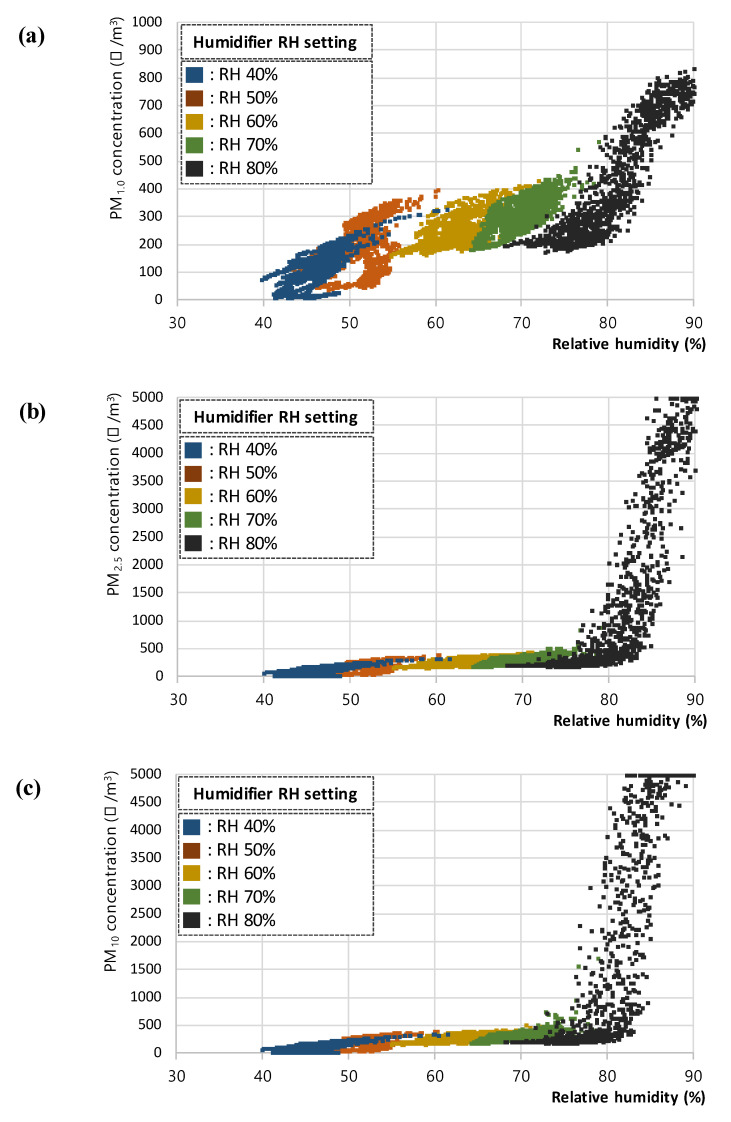
Plots of (**a**) PM_1.0_, (**b**) PM_2.5_, and (**c**) PM_10_ concentration with respect to relative humidity for the steam RH test.

**Figure 5:**
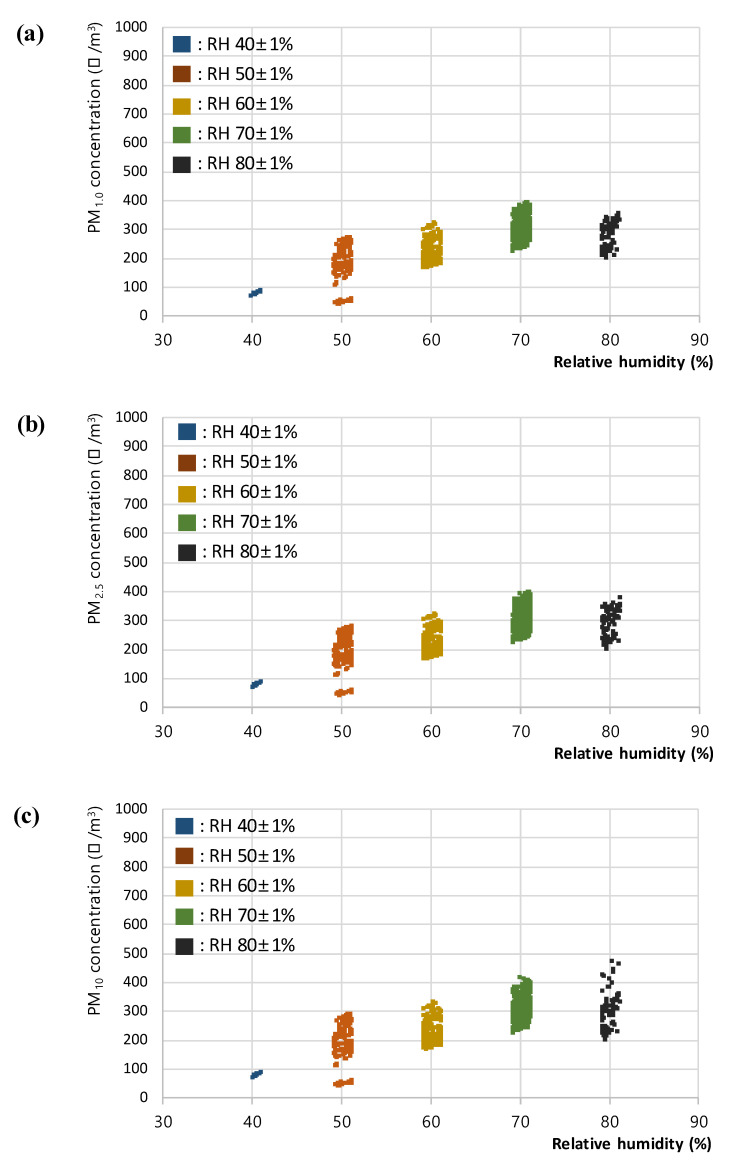
Extracted plots of (**a**) PM_1.0_, (**b**) PM_2.5_, and (**c**) PM_10_ concentration with respect to relative humidity for the steam RH test.

In the steam RH test, the PM concentration was measured according to the RH set by the humidifier, and the results of 7980 data for (a) PM_1.0_, (b) PM_2.5_, and (c) PM_10_, respectively are shown in Figure 4. The steam supply from the humidifier used in this study was continued and discontinued to maintain the set RH; because of this limitation, a constant RH level could not be maintained. Hence, among the collected data, the data within the ±1% range for each RH level were retained and plotted and analyzed. The final set of 1044 extracted data for (a) PM_1.0_, (b) PM_2.5_, and (c) PM_10_, respectively used in the analyses are presented in Figure 5.

In addition, a sudden deviation in concentration was detected for all three PM types: PM_1.0_, PM_2.5_, and PM_10_, in levels above RH 80%. For PM_2.5_ and PM_10_ in particular, the limit of detection of the PM sensor used in this study (5000 μg/m^3^) was measured to assess the difficulty in obtaining normal measurements at RH levels above 80% because of the excess steam in the interior. Accordingly, the standard errors of the PM_1.0_, PM_2.5_, and PM_10_ concentration data in each RH range were analyzed to identify the degree of measurement error. As a result, the standard errors of the PM_2.5_ and PM_10_ data in the RH 80 ± 1% range (33.91 and 87.77, respectively) reached a significantly higher level than the errors in the other RH ranges. Therefore, the outliers were removed from the PM data in the RH 80 ± 1% section; the measured PM concentration data within the normal range were analyzed after excluding the data above RH 80 ± 1%. In addition, the data from the RH 40 ± 1, 50 ± 1, 60 ± 1, and 70 ± 1% ranges were checked for outliers; no further outliers were found, and the standard errors in the PM_2.5_ and PM_10_ data in the RH 80 ± 1% range were lowered to an appropriate level. The standard errors of the PM concentration data in each RH range are presented in Table 2.

### 3.3. Analysis of Test Results

**Figure 6:**
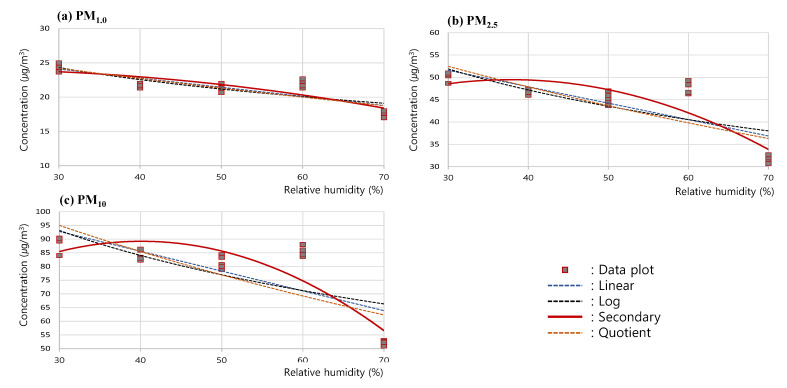
Correlation analysis process of (**a**) PM_1.0_, (**b**) PM_2.5_, and (**c**) PM_10_ concentration for RH test.

To analyze the trend of the changes in measured concentrations according to the changes in RH, a regression curve was drawn for the measured concentrations of PM_1.0_, PM_2.5_, and PM_10_ in the RH 30–70% range, based on the results of the RH test. In this analysis, the linear graph, as well as the log, secondary, and quotient graphs of (a) PM_1.0_, (b) PM_2.5_, and (c) PM_10_, were comparatively analyzed as in Figure 6; the secondary graph exhibiting the highest correlation coefficients, 0.855 (PM_1.0_), 0.869 (PM_2.5_), and 0.883 (PM_10_), was shown to be the one that most reliably expressed the trend of changes in PM concentrations.

**Figure 7:**
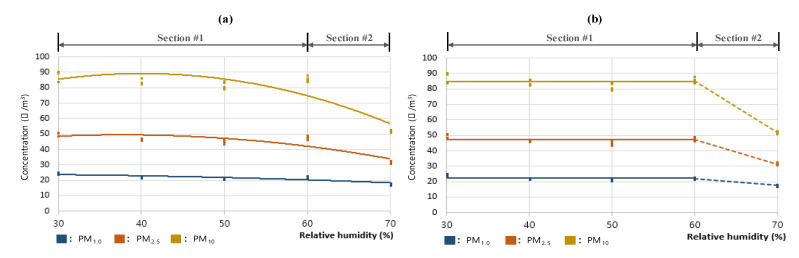
Plots for PM concentration with respect to relative humidity for the three PM types of (**a**) RH-PM#1, which shows the secondary graph, and (**b**) RH-PM#2, which shows the constant PM concentration level below RH 60% and concentration drop above RH 60% in the RH test analysis.

However, as the changes in PM concentrations according to the changes in RH are not significant at levels below RH 60%, the interpretation should presume that the PM concentration is constant below RH 60%. Thus, additional analyses were performed on the secondary curve (a) RH-PM#1 for the PM_1.0_, PM_2.5_, and PM_10_ concentrations; Figure 7 shows the graph (b) RH-PM#2 presuming constant PM concentrations below RH 60%. For this, the RH 30–60% range was set as #1, and the RH 60–70% range was set as #2. Moreover, the average errors across the PM_1.0_, PM_2.5_, and PM_10_ data for Section #1 of RH-PM#1 and RH-PM#2 were comparatively analyzed. The results indicated that for all three PM types, PM_1.0_, PM_2.5_, and PM_10_, RH-PM#2 had lower average errors than RH-PM#1, indicating a higher level of suitability. This implied that the measurements below RH 60% had a constant concentration; further, when the maintained level was approximately 50 μg/m^3^ (for PM_2.5_), that is, the reference concentration in the test, gaseous vapor was determined not to affect the PM concentration measurements by light-scattering sensors. In addition, as previous studies showed that the deposition rate of PM increases when the particle size increases for various reasons, including adsorption [37,38,39], the fall in concentration for the levels above RH 60% was presumed to be because of the adsorption of PM on the condensed water particles at a high humidity level and, thus, should not be interpreted as a measurement error of the light-scattering sensor.

**Figure 8:**
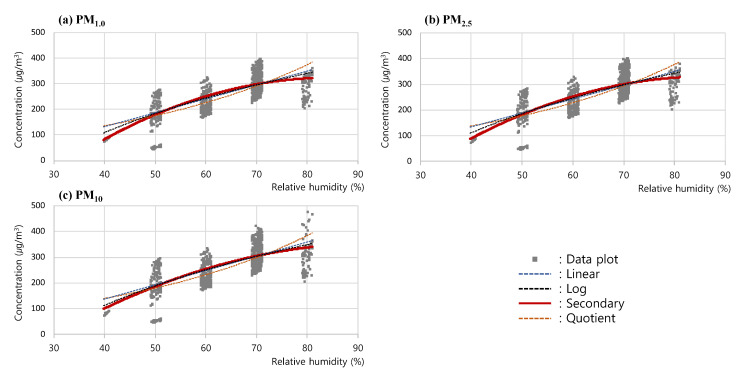
Correlation analysis process of (**a**) PM_1.0_, (**b**) PM_2.5_, and (**c**) PM_10_ concentration for Steam RH test.

Next, to analyze the trend of the changes in measured concentrations according to the changes in steam, a regression curve was drawn for the measured concentrations of (a) PM_1.0_, (b) PM_2.5_, and (c) PM_10_ in the RH 40–80% section, based on the results of the steam RH test as in Figure 8. In this analysis, as with the previous RH test analysis, the linear graph, as well as the log, secondary, and quotient graphs, were comparatively analyzed, and the secondary graph (Steam-PM#1) exhibiting the highest correlation coefficients, i.e., 0.727 (PM_1.0_), 0.722 (PM_2.5_), and 0.709 (PM_10_), was shown to be the one that most reliably expressed the trend of the changes in the PM concentration.

**Figure 9:**
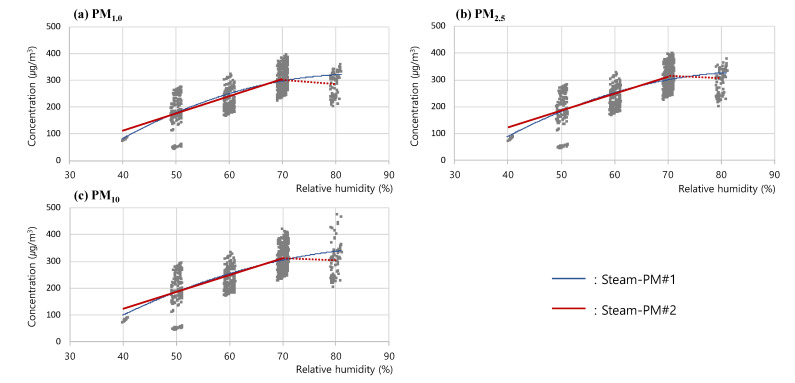
Plots for (**a**) PM_1.0_, (**b**) PM_2.5_, and (**c**) PM_10_ concentration with respect to relative humidity of Steam-PM#1, which shows the secondary graph, and Steam-PM#2, which shows linear increase below RH 70% and concentration drop above RH 70% in the Steam RH test analysis.

However, as the PM concentrations steadily increased according to the changes in RH in the sections below RH 70% with a sudden fall in measured concentrations in the sections above RH 70%, a separate analysis was required for the changes in the measurements for the sections below RH 70%. Thus, an additional regression curve was drawn for the PM concentrations and RH values below 70%. The results indicated that the linear graph (Steam-PM#2) exhibiting the highest correlation coefficients, i.e., 0.767 (PM_1.0_), 0.756 (PM_2.5_), and 0.740 (PM_10_), was the one that most reliably expressed the trend of the changes in the measured concentrations for the sections below RH 70%. This implied that the PM concentrations measured below RH 70% increased at a constant rate as the steam supplied by the humidifier increased at a constant rate. Hence, in contrast to gaseous vapor, steam vapor was determined to have a significant impact on the PM concentration measurements by light-scattering sensors. In addition, in the sections above RH 70%, the walls of the test chamber had excess steam particles combining to flow as condensed water drops, which is presumed to have reduced the number of steam particles counted by the PM sensor and caused the sudden fall in measured concentrations. The results of the steam RH test analysis for (a) PM_1.0_, (b) PM_2.5_, and (c) PM_10_ are presented in Figure 9.

The two tests consequently presented that gaseous humidity does not affect the PM measurement value obtained by the light-scattering sensor, while steam humidity highly increases the measurement value. The results show that the light-scattering sensor could measure the steam vapor aerosols as particles while monitoring dust concentration in ambient air. Since the most representative phenomenon of steam vapor is fog, value correction should be considered for the PM data collected in a foggy environment.

## 4. Discussion

The results in this study suggested that gaseous vapor did not exert an effect on light scattering in the measurement of PM concentrations, whereas steam vapor had a significant impact on the concentrations measured using light scattering. This implied that precautions should be taken regarding measurement errors caused by the fog rather than the humidity fluctuation in the real-time monitoring of PM concentrations in an actual outdoor environment. The significance of this study lies in having identified the key factors to be considered in PM monitoring in outdoor conditions, which have more frequent changes than indoor conditions. Notably, as a global emphasis has been placed on the real-time utilization of urban environmental data using smart technologies such as information and communication technologies (ICT) and the Internet of Things (IoT), the findings in this study are anticipated to serve as the basis to ensure the provisioning of more accurate data on air pollution.

One method of correction for the errors caused by the fog in light-scattering PM sensing is the installation of a heating device in the measuring instrument to remove the fog. Such heating devices are widely used in various conventional outdoor particle counters as the most common method of vapor removal. However, it is difficult to confirm the complete removal of the fog using the heating facility, and there is a problem with the need for periodic maintenance of the heater itself. In addition, due to the installation of the heater, the cost of the instrument is generally increased; the volume and weight are also increased, which imposes limitations on mobile measurements at different points. Notably, when a particle counter is developed, the heating facility is made to operate at all times or operate if the RH exceeds a set level, and in this study, the RH in the absence of fog was found not to influence the PM concentration measurements, indicating that the unnecessary operation of the heating facility could be a factor negatively affecting the power consumption efficiency of the particle counter.

Meanwhile, air quality monitoring has become a very concerning issue among people because of the negative effects of air pollution on health [8]. There has been a growing demand for real-time sensing, which could monitor air pollution in wide areas more precisely [40]. As a result, low-cost light-scattering PM sensors, suitable for developing IoT-based monitoring networks, have appeared and have been widely spread [40]. Since each low-cost sensor performs a role of a single node in a wide network, the accuracy of monitoring networks highly depends on the performance of the sensor applied.

Consequently, considering the growing use of low-cost light scattering sensors, there is a need for the development of a novel steam removal technology to complement the drawbacks of such heating facilities or an error correction technology for the PM concentration data according to the fog concentration. This requires a more precise measurement of the steam aerosol concentration beyond RH, and further studies should investigate the effects of the steam aerosol concentration on light-scattering PM sensing through the use of RH sensors as well as fog sensors.

Moreover, temperature could also be an important factor that affects the PM concentration measurement because RH is a percentage that represents the amount of water vapor in the air at a given temperature when compared with the maximum possible water vapor at that same temperature. The amount of maximum possible saturated water vapor depends on the ambient temperature. This implies that RH could change as the temperature changes, even though the absolute amount of vapor in the ambient air is steady. Meanwhile, the air pressure could also be an important factor that could affect the RH control because RH is a value of water vapor pressure divided by saturation water vapor pressure.

However, the temperature and the air pressure were assumed as constant values in both the RH test and steam RH test without considering them as variables. Therefore, additional tests to investigate the effect of temperature and pressure on PM concentration are required to support a more precise PM measurement in further studies.

## 5. Conclusions

This study aimed to determine the effects of the humidity state on the measurements using a light-scattering PM sensor, and thus, tests to analyze the changes in the PM concentrations measured after supplying gaseous and steam vapors were conducted. The results of these tests led to the following conclusions:

(1) To determine the effects of gaseous humidity on the measurements using the light-scattering sensor, an RH test with the supply of gaseous vapor was conducted. As a result, the concentration data for PM_1.0_, PM_2.5_, and PM_10_ were collected according to the changes in RH in the RH 30–70% section. The collected data were analyzed to show that the PM concentrations were at a constant level in the sections below RH 60%, while in the RH 60–70% section, the adsorption of PM by the condensed water particles in high humidity led to a sudden fall in PM concentrations.

(2) To determine the effects of humidity in liquid aerosol on the measurements by the light-scattering sensor, a steam RH test with the supply of steam vapor using an ultrasonic humidifier was conducted. The results indicated a linear increase in the PM concentrations according to the RH increasing with the steam supply in the sections below RH 70%, while in the sections above RH 70%, the steam particles combined with one another to form water drops, causing a fall in PM concentrations.

(3) Based on the findings, gaseous humidity was determined not to cause errors in the PM measurements via the light-scattering method. In contrast, steam humidity, such as that caused by fog, was determined to cause errors, as it is included in the PM counting.

(4) In the conventional outdoor PM measurements using a light-scattering sensor, such errors are corrected by removing the fog using a heating facility installed on the measuring instrument. This, however, entails several technological limitations, which should be overcome through the development of a novel steam removal technology or a data correction technology with a focus on the fog concentration beyond RH.

## Figures and Tables

**Table 1:** PM concentration data for the RH test in the RH 30–70% range.

RH (%)	MI	PM Concentration Data (μg/m^3^)														
		PM_1.0_					PM_2.5_					PM_10_				
30	MI 1.	24	24	25	26	25	49	49	49	49	49	83	82	84	83	81
	MI 2.	27	27	29	29	28	47	46	48	48	48	75	74	74	73	74
	MI 3.	21	20	21	20	20	55	51	56	55	56	113	96	110	112	116
	Avg.	24	24	25	25	24	50	49	51	51	51	90	84	89	89	90
40	MI 1.	22	22	21	22	22	46	45	45	45	45	82	80	80	80	80
	MI 2.	26	26	25	25	25	44	43	42	42	42	68	66	65	68	70
	MI 3.	18	17	18	18	18	52	50	53	51	53	108	101	105	101	109
	Avg.	22	22	21	22	22	47	46	47	46	47	86	82	83	83	86
50	MI 1.	21	22	22	22	22	43	46	45	45	45	77	80	79	79	79
	MI 2.	24	26	26	25	25	42	41	42	42	43	71	61	63	64	69
	MI 3.	17	17	18	19	17	46	47	49	54	50	94	96	98	110	102
	Avg.	21	22	22	22	21	44	45	45	47	46	81	79	80	84	83
60	MI 1.	22	22	21	23	24	46	46	45	47	49	81	81	80	82	81
	MI 2.	24	26	26	26	27	43	45	45	44	46	73	74	73	69	72
	MI 3.	18	18	18	18	17	50	49	50	54	53	97	99	100	107	111
	Avg.	21	22	22	22	23	46	47	47	48	49	84	85	84	86	88
70	MI 1.	19	19	19	20	19	33	31	33	35	33	55	49	52	58	52
	MI 2.	19	20	18	20	20	30	30	28	31	32	47	47	45	48	50
	MI 3.	14	14	14	14	15	31	31	31	30	33	57	57	56	52	55
	Avg.	17	18	17	18	18	31	31	31	32	33	53	51	51	53	52

Notes: MI: Measuring instrument; Avg.: Average value.

**Table 2:** Standard errors of PM concentration data in steam RH test in RH 40 ± 1%, 50 ± 1%, 60 ± 1%, 70 ± 1%, and 80 ± 1% ranges.

PM	Standard Error of PM Concentration Data (μg/m^3^)					
	RH 40 ± 1%	RH 50 ± 1%	RH 60 ± 1%	RH 70 ± 1%	RH 80 ± 1%	
					Before Data Extraction	After Data Extraction
Number of data (PM_1.0_, PM_2.5_, PM_10_, respectively)	12	186	239	534	152	73
PM_1.0_	1.45	4.57	2.34	1.72	7.84	5.22
PM_2.5_	1.49	4.71	2.36	1.76	33.91	5.44
PM_10_	1.53	4.91	2.42	1.86	87.77	7.51

## Data Availability

The data used to support the findings of this study are available from the corresponding author upon request.
